# The impact of Kinesio taping technique on children with cerebral palsy

**Published:** 2016-10-07

**Authors:** Alireza Shamsoddini, Zabihallah Rasti, Minoo Kalantari, Mohammad Taghi Hollisaz, Vahid Sobhani, Hamid Dalvand, Mohammad Kazem Bakhshandeh-Bali

**Affiliations:** 1 Exercise Physiology Research Center, Baqiyatallah University of Medical Sciences, Tehran, Iran; 2 Department of Occupational Therapy, School of Rehabilitation, Shahid Beheshti University of Medical Sciences, Tehran, Iran; 3 Department of Physical Medicine and Rehabilitation, School of Medicine, Baqiyatallah University of Medical Sciences, Tehran, Iran; 4 Department of Occupational Therapy, School of Rehabilitation, Arak University of Medical Sciences, Arak, Iran; 5 Pediatric Neurology Center of Excellence, Department of Pediatric Neurology, Mofid Children Hospital, School of Medicine, Shahid Beheshti University of Medical Sciences, Tehran, Iran

**Keywords:** Balance, Cerebral Palsy, Hand Function, Kinesio Taping, Motor Function

## Abstract

Cerebral palsy (CP) is the most common movement disorder in children that is associated with life-long disability and multiple impairments. The clinical manifestations of CP vary among children. CP is accompanied by a wide range of problems and has a broad spectrum. Children with CP demonstrate poor fine and dross motor function due to psychomotor disturbances. Early rehabilitation programs are essential for children with CP and should be appropriate for the age and functional condition of the patients. Kinesio taping (KT) technique is a relatively new technique applied in rehabilitation programs of CP. This article reviews the effects of KT techniques on improving motor skills in children with CP. In this study, we used keywords "cerebral palsy, Kinesio Tape, KT and Taping" in the national and international electronic databases between 1999 and 2016. Out of the 43 articles obtained, 21 studies met the inclusion criteria. There are several different applications about KT technique in children with CP. Review of the literature demonstrated that the impact of this technique on gross and fine motor function and dynamic activities is more effective than postural and static activities. Also this technique has more effectiveness in the child at higher developmental and motor stages. The majority of consistent findings showed that KT technique as part of a multimodal therapy program can be effective in the rehabilitation of children with CP to improve motor function and dynamic activities especially in higher developmental and motor stages.

## Introduction

Cerebral palsy (CP) is a neurological non-progressive disorder resulting from brain damage occurring before, during, or after birth^1^^,^^2^ along with permanent disorder of movement and posture.^3^ It is the most common movement disorder associated with lifelong disability and motor deficit.^4^ The topographic classification of CP is hemiplegia, diplegia, and quadriplegia. Another classification is based on motor function as pyramidal (spastic) and extrapyramidal (non-spastic including athetoid, ataxic, and dystonic). The prevalence of CP is about 2 to 2.5 per 1000 live births.^3^^,^^5^ According to the International Classification of Functioning system (ICF), CP affects the body structures (e.g. limbs), body function (e.g. intellectual function), activities (e.g. standing/walking), and participation (e.g. sport). These deficits subsequently lead to some disabilities including impairments, limitation in function, and restriction in participation.^6^ Psychomotor disturbances in children with CP results in limitation in use of the limbs, more paralysis, difficulty in performing activities of daily living (ADL), more dependence and ultimately lower quality of life. Therefore, it is essential that the treatment be provided early and effectively.^7^^,^^8^ A variety of commonly therapeutic options are used for CP treatment including botulinum toxin injection,^9^ orthopedic surgery, Constraint-induced Movement Therapy (CIMT),^10^ oral medications,^10^ occupational and physical therapy.^11^^-^^14^ The aim of occupational and physical therapy in the treatment of children with CP is to normalize the muscle tone**, **reduce the muscle and joint contractures and improve the sensory and cognitive problems, improve muscles strength, increase the range of motion (ROM) and fostering children's independence level in ADL^15^^-^^17^ by means of a number of various dynamic approaches including Bobath,^18^ Sensory Integration (SI), proprioceptive neuromuscular facilitation (PNF)^19^ and the Brunnstrom techniques.^3^^,^^18^ Kinesio taping (KT) is a relatively new therapeutic tool used in rehabilitation program of children with cerebral palsy, although it has been used for a long time in sport or orthopedic fields, and has been approved as a supplemental intervention for other functional impairments.^20^^-^^23^ Kinesio tape is a specialized elastic-like tape made of latex-free cotton fibers having no medication effect^24^ and designed to mimic the elasticity properties of the muscle, skin and fascia.^23^ By proper taping, the elasticity of the tape not only does not restrict the soft tissue, but also supports the weak muscles and creates a full ROM. It has been hypothesized that KT may favorably stimulate the coetaneous receptors of the peripheral sensorimotor system, since these receptors are associated with pain, proprioception and motor control.^25^ Taping can influence the skin, lymphatic system, circulatory system, fascia, muscle and joint^26^ and theoretically leads to enhancing proprioception,^27^ diminishing pain and edema, reducing muscle spasms, and strengthening the muscles.^28^^,^^29^ KT supports the joints by correcting the muscle function, restoring the proprioception, optimizing the postural alignment and stimulating the coetaneous receptors. It can reduce the pain and provide the proprioception feedback for reaching and maintaining the natural body posture as well.^30^^-^^33^ KT application, in conjunction with other regular rehabilitation programs for the children with CP, may positively influence the sensorimotor system resulting in improved voluntary control and coordination of the upper-limbs.^21^^,^^31^^,^^32^ Given the above evidences and the importance of the treatment in children with CP, in addition to investigating KT as a new therapeutic intervention, the main purpose of this study is to have a review in order to evaluate the effectiveness of KT in neurorehabilitation of the children with CP. Another purpose of the present study is to collect the existing literature dealing with Kinesio tape in a single article, to analyze the results and finally to reach the overall conclusion.

## Materials and Methods

Nine electronic databases were searched: PubMed, Google Scholar, Science Direct, Ovid, Scopus, Proquest, Web of Knowledge, CINAHL and Islamic World Science Citation Center (ISC) from earliest records to December 2015. Existing systematic reviews and major publications on KT Technique in children with CP were sourced to identify appropriate search terms. Search terms included 'CP', 'taping', 'Kinesio tape', and 'KT'. The references of the papers were also manually searched in order to identify the other potentially eligible studies. An initial review was undertaken of all titles and abstracts. All articles considered appropriate were read in full to establish if they met the eligibility criteria. Inclusion criteria were: 1) the availability of abstract or full text of the articles; 2) the studies were merely conducted on CP and KT. Studies were excluded if children with CP had received botulinum toxin injection prior to the intervention or as part of the treatment or comparative therapy.

## Results

**Figure 1:**
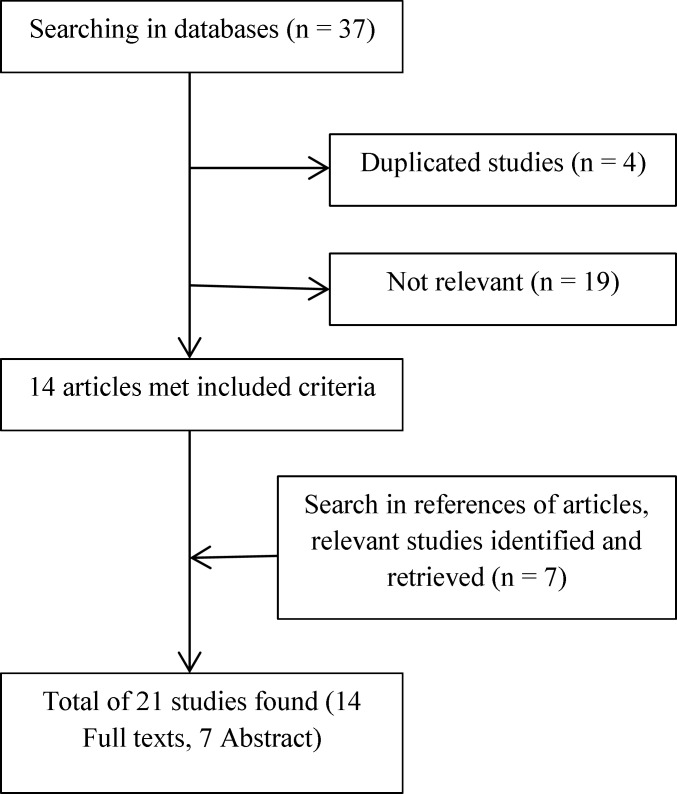
Flowchart of study identification

A flow chart of the selection process is shown in figure 1. After conducting the searches based on the inclusion and exclusion criteria, a total number of 37 articles were collected; out of which 21 articles, including 14 full text articles and 7 abstracts, fulfilled the inclusion criteria. Among the 21 selected articles conducted on the effects of KT in the children with CP; eight studies were conducted on the hand and upper-limb, six studies on the lower limb, five studies on the trunk and vertebral column, one study performed on drooling and only one was a commentary article. A summary of all articles included in this review can be found in table 1.

## Discussion

According to the reviewed papers, we have found that KT can be used in rehabilitation in combination with other common therapeutic techniques including: increasing of strength, enhancement of endurance, improving ROM, and reduction of spasticity. For a better understanding; papers were examined in three following sections:

***Hand and upper extremity***

The results of all studies that investigated the effectiveness of KT on the hands and upper extremity were significantly positive; and the authors concluded that KT technique could be effective for improving the upper extremity motor skills in the children with CP. In the articles reviewed, the purposes of KT application on the upper-limb were: 1) positioning of the wrist, palm, and thumb in the functional position (opening the hand to sensorial stimulate and improve the function of the thumb and other fingers);^12^^,^^34^^-^^38^ 2) reducing the spasticity;^38^ 3) forearm supination;^12^ 4) maintaining the shoulder in a functional position;^34^ and 5) promote active ROM of wrist and fingers.^35^^,^^36^^,^^39^ In all relevant papers, the authors have described the benefits of using KT, although Keklicek et al.^37^ believed that no direct correlation exists between the spasticity and the functional ability, and the improvement in Modified Ashworth Scale does not necessarily leads to a more functional use of the upper-limb. The results of the identified literature shows that KT applied in hand and upper-limb may result in enhancing the motor function, timing, speed and smoothness of the movement, active ROM, dexterity, grasp and release as well the spasticity reduction, but it has no effect on weight bearing and protective extension in children suffering from spastic diplegic CP.^12^^,^^34^^,^^37^^,^^38^ Tape can promote wrist active ROM; whatever, in studies of Demirel and Tunay^36^ and Bahadir Agce et al.^39^ these change were significant, but in study of Chitaria et al.^35^ these were not significant; variances in results could be due to differences in intervention period of these studies.

***Trunk/lower extremity***

In the studies that investigated the effect of KT on gross motor skills and functional abilities in trunk and lower extremity, it was applied on ankle (in order to reduce spasticity of Achilles and increase the strength of the tibialis anterior), back of the knee (controlling the genus recurvatum), para spinal muscles, and quadriceps muscles. In the published studies conducted on the lower extremity, improvement in gross motor function, dynamic activities, trunk and posture control, and muscle balance in sitting and standing position were reported.^32^^,^^33^^,^^35^^,^^36^^,^^39^^,^^41^ Conversely, da Costa et al.^30^ found no direct effect in the static activities after using KT. Also despite improving in motor skills, Iosa et al.^40^ did not find any change in the modified Ashworth scale and equinus foot. The results revealed that KT is an effective method in dynamics activities, like sit-to-stand, walking and movement patterns, improving in ADL and ROM, and spasticity reduction,^30^^,^^33^^,^^40^^-^^42^ but not effective in static balance and static postural control.^30^ Moreover, no significant changes were found in GMFM score and sitting posture of quadriplegic children at levels 4 and 5 of GMFCs scale.^41^ 

**Table 1:** A summary of the examined articles (the arrangement based on taping area and the articles full text or the abstracts)

**Author**	**Objectives**	**Type of study**	**Sample size**	**Age (year)**	**Area taped**	**Outcome measure**	**Intervention period**	**Results**
Chitaria et al.^35^	Evaluate short-term effects of KT on fine motor function and active wrist extension ROM in CP	Quasi-experimental	15	3-6	Lateral epicondyle of the humerus to dorsal aspect of metacarpal head	PDMS-2, Video recording (for AROM)	3 days	Significant changes were found in fine motor. AROM of wrist extension changed but these were not significant.
Keklicek et al.^37^	To investigate the effect of tape application on thenar, palmar and upper limb of children with CP	RCT	45	4-14	Extensor surface of the thumb and first web space	NHPT, NPPT	20 Minutes	Significant difference between groups and positive effect of KT on the hand function.
Demirel and Tunay^36^	Determine effect of Kinesio tape on active ROM of the wrist	Pilot study	15	6-18	Extensor muscle of wrist	Goniometer	45 minute	Statistically significant changes were found in wrist extension, radial, ulnar deviation AROM and wrist extension ROM while functional ball grasping.
Camerota et al.^34^	To investigate the influence of NMT on the upper limb in a child with left hemiplegia CP	Case study	1	17	Palmar, cervical, anterior & posterior region of shoulder	3D movement analysis	15 days and exchanging the tape each 3 days	Improvement in movement duration, average movement jerkiness, movement speed & smoothness, ROM and less segmented movement.
Sadeghi Moghaddam et al.^38^	To study the effects of KT on wrist in spastic diplegic CP	RCT	26	3-6	Extensor surface of wrist	QUEST, MAS	12 days new taping each 3 days	Spasticity reduction, improvement in grasps and dissociated movements of fingers; no significant differences were found in weight bearing and protective extension.
Mazzone et al.^12^	To assess the effectiveness of KT applied to upper-limb of Hemiplegic CP	Pilot study	16	3 ± 2	Thumb (for extension), forearm (for supination)	Melbourne	17 Months (7 months in the middle of the protocol without taping)	Eight out of the 16 participants completed the entire protocol. Significant difference in the result of all participants.
Bahadir et al.^39^	Analyse the effect of wrist correction Kinesio tape on hand span in CP	Experimental	7	6.78 ± 2.7	Dorsum aspect of wrist and finger	Goniometer	Immediate	Wrist extension angle significantly increased after application.
Demirel^51^	To study KT effects on grasping and release	Experimental	25	Mean: 10	The palm, the first web space, and dorsum of the hands	MACS, MAS	-	Positive result in all variables test.
Ibrahim^44^	Investigate the effect of Kinesio tape on the trunk in spastic diplegic CP	RCT	30	7-10	erector spine muscles from S1 to C7	GMFM-88, PBBS, Formetric instrumentation system	12 weeks (changing tape every 3 days with a day break)	Sitting control, postural parameters, standing control and balance were significantly changed in both groups; but treatment group was more significantly changed than the control group. in pelvic torsion and surface rotation, there were no significant change.
Simsek et al.^33^	To study the effects of KT on sitting posture, gross motor function and functional independence in CP	RCT	31	8 ± 4	Para spinal S1- C7	GMFM, WeeFIM, SAS	12 weeks (changing tape every 3 days with a day break)	Positive effect on sitting posture, no direct effects on gross motor function and functional independence.
Footer^41^	To assess therapeutic taping effectives on dysfunctional sitting and control gross motor function in quadriplegic CP	RCT	18	3-13	Para spinals	GMFM-88	12 weeks	No significant differences were found for the GMFM scores.
Elbasan Uzun Akkaya^46^	Investigate the effects of NMES and KT in addition to NDT, on sitting balance in CP	Crossover, before-after trial	4	5-12	Paravertebral muscles	MMT, GMFCS, GMFM, SPCM, Modified functiona reach, WeeFIM, CP QOL	6 weeks	Significantly change was found in abdominal and trunk extensor muscle strength, GMFM, CP QOL and functional reach test Combination of KT, NMES and NDT is more effective than each one.
Burditt^45^	The effect of KT on dysfunctional sitting control in quadriparesis CP	RCT	18	-	-	EMG, Kinematic, GMFM	12 weeks	Differences in the GMFM and EMG of the Para spinal musculature were NOT significant.
Kaya et al.^50^	To evaluate activity and body function of hemiplegic CP	RCT	30	7-14	Ankle, knee, hip, trunk, shoulder, forearm and wrist	WeeFIM, BOTMP, GMFM, Short-term muscle power	12 weeks (taping 6 days per week)	Positive results in all assessment tests.
da Costa et al.	To assess the immediate effects of KT on STS, balance and dynamic postural control in CP	Pilot study	4	9-11	Quadriceps and tibialis anterior	Motion analysis, PBS, TUG	1 day	Positive results in two tests STS and TUG, no difference in PBS score.
Ghalwash et al.^47^	Investigate the effect of adhesive taping in controlling genu recurvatum in diplegic CP	RCT	14	5-7	Back of the knee (thigh and calf) with x pattern	GMFM-88, Auto-CAD, Screen protractor	12 weeks (changing tape every 60 hours)	No significant changes were found.
Iosa et al.^40^	To promote the developmental motor stage (investigation the KT technique as a non-invasive method) in hemiplegic CP	Experimental	8	Mean: 5	Ankle, knee and hip if necessary	MAS, GMFM, Goniametery, Gait analysis	12 months (the first 6 months physiotherapy alone, and the next 6 months combined with KT)	Function improving (increase in GMFM score and walking speed), improved stability (decrees in the step width and back knee), improving in limb symmetry and movement pattern; no change in Ashworth score, ROM and equines.
Greve et al.^52^	To reduce the spasticity in diplegic CP	Case study	1	4	Ankle	EMG, ROM, MAS	26 days	Positive change of EMG in tibialis anterior and Triceps Surae, and spasticity reduction in the gastrocnemius.
Iosa et al.^42^	To improve gait hemiplegic CP	Pilot study	2	7 and 10	Ankle	Gait analysis	6 months (wearing the tape 6 days per week)	Gait with normal ankle and less back knee due to reduction in spasticity.
Nieves Estrada et al.^49^	To compare the efficacy of electrical stimulation and KT on drooling in CP	Quasi-experimental	18	-	-	Frequency and severity of drooling	-	Both interventions had equal positive effect.
Iosa^43^	Commentary on the study done by Kaya in 2014	Review	1. KT is an important step in neurorehabilitation program of children with CP 2. KT technique is more effective at levels I, II, GMFM 3. KT in dynamic activities is more effective than static activity 4. KT technique can encourage the children to use their few available resources.					

NMT: Neuromuscular Taping; KT: Kinesio taping; CP: Cerebral palsy; RCT: Randomized control trial; ROM: Range of motion; NDT: Neurodevelopmental treatment; STS: Sit-to-stand

Therefore, KT seems to be more beneficial at the levels 1 and 2 GMFCs and also in dynamic activities. Moreover, taping may encourage less-involved children to use their affected limbs for the maximum ability; however, it is not effective in children with severe involvement. A study revealed that the dynamic activities require more postural control than the static activities,^43^ but, in another study KT influenced the dynamic activities while had no effect on the static activities.^30^ Ibrahim^44^ found that KT significantly improved sitting control, postural parameters, standing control and balance; but in pelvic torsion and surface rotation there were no significant change. Although other studies have examined the effect of KT on trunk and paraspinal muscles, only in one study sitting posture had positive change.^34^ In these studies, authors found no significant change in the GMFM score and functional independence.^33^^,^^41^^,^^45^

In study by Elbasan and Uzun Akkaya^46^ that compared the effect of three techniques in three groups (group 1, neurodevelopmental treatment (NDT); group 2, NDT + KT; group 3, NDT + KT + neuromuscular electrical stimulation (NMES), the results showed that the combination of all these modalities (group 3) is more effective on abdominal muscles and trunk extensors, trunk control and posture, functional reach, and ADL that finally led to promotion of quality of life in children with CP and family of them. In a study aimed to promote motor development in children with hemiplegia CP by use of KT on the ankle, authors found the positive influence on the functional skills, walking, symmetrical limbs and locomotor in all participants except one case who also had dyspraxia with SI dysfunction. Therefore, the authors concluded that, in this one exceptional case, it was less likely that child could properly express the increase in the sensory feedback. Furthermore, in spite of the favorable change in functional movements, no significant change occurred in spasticity.^40^ This is consistent with of Keklicek et al.^37^ finding that showed the spasticity is not directly related to functional ability. Also, these results indicate the opposite effect of the serial casting where gradual reduction in spasticity and ROM increment occurs, without improvement in functional activity.^37^^,^^40^ Only in one study, the effect of adhesive tape on genu recurvatum in diplegic spastic CP were investigated, but results showed no significant difference.^47^ Authors noted that these results may be due to limited ability of tape to overcome the musculoskeletal problem. In this study, value of GMFM was improved that could be attributed to tape's pressure or traction on the skin which provides cutaneous sensory stimulations, so more proprioceptive input passed to the central nervous system. In taping group, joint protection and support provided by the tape could also be another reason of significant improvement in ability of standing and walking.^47^

***Others***

Drooling is a common problem in children with CP. Cause of these problem is insufficient lip closure and impairment in tongue movements due to diminished sensory perception in oral and perioral.^48^ Nieves Estrada et al.^49^ compared the effectiveness of KT and NMES techniques on drooling. The results showed that two interventions are equally effective on drooling. 

## Conclusion

Bearing in mind the results of these studies, especially the considerable results of those by Kaya et al.^50^ and Keklicek et al.^37^ and Ibrahim,^44^ these can be concluded that KT favorably impacts the fine and gross motor abilities and functional independence in ADL, sitting/standing control and balance, etc. Based on these studies, KT is more effective in mild to moderate CP and is not effective in severe CP. Psychological effect of KT can encourage children to fully use their limited ability. The important point about KT is to be used in adjunct with other rehabilitation techniques. This result may influence the therapists’ decision to apply KT in neurorehabilitation program for the children with CP. One of the limitations of this study was the small number of relevant published studies. Another limitation was that most of the authors had not mentioned the method of applying KT on the body areas. Therefore, we could not find any relation between the method of taping and the effects of KT. For more accurate results, comparing the effects of this technique with the other rehabilitation techniques in the children with CP, in addition to investigating the efficacy of KT intervention in other neurological diseases, such as stroke, is recommended.

## Notes:

**How to cite this article: **Shamsoddini A, Rasti Z, Kalantari M, Hollisaz MT, Sobhani V, Dalvand H, et al. The impact of Kinesio taping technique on children with cerebral palsy. Iran J Neurol 2016; 15(4): 219-27.
